# Unattended and attended visual change detection of motion as indexed by event-related potentials and its behavioral correlates

**DOI:** 10.3389/fnhum.2013.00476

**Published:** 2013-08-14

**Authors:** Nele Kuldkepp, Kairi Kreegipuu, Aire Raidvee, Risto Näätänen, Jüri Allik

**Affiliations:** ^1^Institute of Psychology, University of TartuTartu, Estonia; ^2^Doctoral School of Behavioural, Social and Health Sciences, University of TartuTartu, Estonia; ^3^Center of Integrative Neuroscience, University of AarhusAarhus, Denmark; ^4^Cognitive Brain Research Unit, Institute of Behavioural Sciences, University of HelsinkiHelsinki, Finland; ^5^Estonian Academy of SciencesTallinn, Estonia

**Keywords:** visual mismatch negativity (vMMN), attention, oddball paradigm, motion detection, event-related potential (ERP)

## Abstract

Visual mismatch negativity (vMMN) is a negative-going component amongst cognitive event-related potentials. It reflects an automatic change-detection process that occurs when an infrequent stimulus is presented that is incongruent with the representation of a frequent (standard) event. In our research we use visual motion (more specifically motion direction changes) to study vMMN. Since movement in the visual field is quite irresistible to our brain, the question in hand is, if the detection of motion direction changes is dependent on attention directed to the stimulus. We present a new continuous whole-display stimulus configuration, where the attention capturing primary task of motion onset detection is in the central part of the visual display and visual oddball sequence on the background. The visual oddball paradigm consisted of 85% standard and 15% deviant events, motion direction change being the deviant. We show that even though the unattended visual oddball sequence does not affect the performance in the demanding behavioral primary task, the differences appearing in that sequence are noticed by our brain and reflected in two distinguishable vMMN components in occipital and parietal scalp locations. When attention is directed toward the visual oddball sequence, we only see different processing of standards and deviants in later time-windows and task-related activity in frontal scalp location. Our results are obtained under strict attention manipulation conditions.

## Introduction

It is both necessary and possible for the human visual system to quickly and effectively detect sudden changes in the visual field even if those changes appear in the visual periphery or attention is not directed to them. This automatic change-detection mechanism has been shown to exist by means of a visual mismatch negativity (vMMN) component of the event-related potentials (ERPs). As its auditory counterpart (auditory MMN, Näätänen et al., 1978; for reviews see Näätänen and Winkler, 1999; Näätänen et al., 2007), vMMN component is elicited by infrequent visual stimuli (i.e., deviants) in the stream of frequent stimuli (i.e., standards) that obey some sequential regularity. It has a negative deflection and usually peaks around 150–400 ms after the onset of a visual stimulus. Researchers have argued that vMMN is elicited when an infrequent stimulus is incongruent with the sensory memory trace of a frequent stimulus (a memory-mismatch account) and that based on the regularities in the preceding stimulus sequence an incongruous prediction is made for the upcoming stimulus (a prediction-error account) (for reviews see Pazo-Alvarez et al., 2003; Czigler, 2007; Kimura et al., 2011; Kimura, 2012).

Proofs for the existence of vMMN remained elusive for some time and only relatively recently solid evidence started to accumulate that MMN exists not only in auditory but visual system as well. Up to now, vMMN has been obtained to differences in several visual features, such as stimulus color (Czigler et al., 2002, 2004; Clifford et al., 2010), location (Berti and Schröger, 2004, 2006), luminance (Stagg et al., 2004), orientation (Astikainen et al., 2004, 2008; Kimura et al., 2009 for left/right hands with different orientation see Stefanics and Czigler, 2012), spacial frequency (Kenemans et al., 2010; Sulykos and Czigler, 2011), duration of the visual stimulus (Qiu et al., 2011), motion direction changes (Lorenzo-López et al., 2004; Pazo-Alvarez et al., 2004a; Kremláček et al., 2006; Amenedo et al., 2007), as well as more abstract sequential regularities (Stefanics et al., 2011; Kimura et al., 2012), object formation (Müller et al., 2010) or deformation (Besle et al., 2005) and stimuli carrying emotional content (Zhao and Li, 2006; Astikainen and Hietanen, 2009; Kimura et al., 2012; Stefanics et al., 2012). As Sulykos and Czigler (2011) have already pointed out, a vast majority of vMMN studies have concentrated on the automatic processing of features that are supposed to be processed by the parvocellular system. With this current study we investigate the change-detection processes in motion perception which is typically thought as a domain of the magnocellular system. Low-level motion perception is widely recognized as a vital function of the visual system and changes in speed and direction of motion are processed automatically without a necessary involvement of the focused attention (Cavanagh, 1992). Therefore, it could be a useful tool to investigate automatic change detection.

One of the main characteristics of the MMN component is its independence of attention: the magnitude of MMN can be approximately the same irrespective of the signal being attended or not (for auditory modality see Näätänen et al., 2007; for visual modality see Pazo-Alvarez et al., 2003; Kimura et al., 2009). Thus, when applying an experimental paradigm to elicit vMMN, the visual stimuli forming deviants and standards are usually task-irrelevant and there is a behavioral primary task that has to capture the subject's attention. To study automatic change detection in auditory modality, multimodal studies are often conducted, using a visual primary task [see Escera and Corral (2007) for some examples]. There have been studies investigating the intermodal effects of stimulation, showing that the amplitudes of ERPs are enhanced to stimuli in the attended modality (Alho et al., 1992; Wei et al., 2002). The stimulation and focused attention in one sensory modality has the capacity to affect perceptions in another modality (Besle et al., 2005; Bendixen et al., 2010; Salminen et al., 2013) and auditory and visual sensory memory are not completely differentiated from each other. Also, Czigler (2007) has pointed out that visual primary tasks guide attention more effectively than auditory, the latter becoming background stimuli too easily in case of continuous stimulation. So while for vMMN studies the primary task sometimes is a task in the auditory modality (e.g., listening to some story or radio play, or reacting to specific sounds: Astikainen et al., 2004; Maekawa et al., 2005, 2009; Fisher et al., 2010), a majority of studies have applied the vMMN paradigm and the primary task both in visual modality. One of the approaches is to use a sequence of stimuli where occasional stimuli function as targets and a behavioral task is related to them (e.g., subjects have to give a manual reaction whenever the targets appear in between the standard and deviant stimuli or when stimuli carrying standard or deviant properties also have target properties: Tales et al., 1999; Berti and Schröger, 2004, 2006; Kimura et al., 2009; Berti, 2011). A step forward is to have a stimulus sequence, where target stimuli are presented in the central part of the visual field and standards and deviants in the periphery (Lorenzo-López et al., 2004; Pazo-Alvarez et al., 2004a; Kremláček et al., 2006). The question is whether there is no attention directed to the non-target stimuli in such sequential stimulus presentations where the stimuli are separated in time [that has also been critically raised by Czigler (2007)]. To take this issue under control, it is rather common to use a central primary task, while at the same time vMMN-eliciting stimulus sequences appear in adjacent locations or visual periphery (some examples of the different stimuli used: Müller et al., 2010; Qiu et al., 2011) and the time-course of stimulus presentation of the two areas is not connected. It has been found though, that vMMN amplitudes for stimuli presented in lower and upper visual hemifield differ (being higher in the lower visual hemifield) (Czigler et al., 2004; Amenedo et al., 2007; Sulykos and Czigler, 2011; Müller et al., 2012; for motion onset evoked potentials see Kremláček et al., 2004). This discrepancy has not been shown for horizontal hemifield locations (Pazo-Alvarez et al., 2004b). The issue of stimulus location has been lately critically raised by Müller et al. (2012), who argue that the block-wise stimulus presentation in lower/upper hemifields does not rule out attention shifts to task-irrelevant stimuli. Derived from the studies indicating vMMN differences due to stimulus presentation location, we propose an experimental design that uses a central primary task and for standard and deviant stimulus presentation the whole peripheral visual field, which should eliminate the exogenous location effects.

The relative motion between an observer and the visual scene creates optic flow which is monitored with a purpose of guiding locomotion (Gibson, 1950). It is very likely that changes in the optic flow pattern are detected automatically at a relatively low level of processing and do not require focused attention for noticing them. The main goal of this study is to investigate the processing of changes in motion flow direction in conditions either requiring focused attention or not. It is predicted that unexpected changes in the flow pattern elicit a vMMN response which magnitude is nearly identical irrespective of attention paid to that change. The observer's task was to detect motion onset of a central area which was surrounded by a peripheral area filled with a horizontally moving pattern. The peripheral area was moving independently of the central one and an oddball paradigm was applied there to elicit vMMN. In an attention neutral task the observer was asked to execute a simple reaction as soon as the central target started to move. In an attention demanding task the observer was instructed to press one of two keys dependent of the relative motion direction between the central and peripheral moving patterns. Since one of the main properties of the MMN is attention-independence (Näätänen et al., 2007) it is expected that vMMN elicited by the peripheral flow pattern is independent of attention allocated to it.

## Materials and methods

### Participants

Forty-nine volunteer observers (mean age 21.2 ± 2.3 years, 14 male) took part in the experiment. They all had normal or corrected-to-normal vision. The participants signed a written consent and the study was approved by the Research Ethics Committee of the University of Tartu [based on The Code of Ethics of the World Medical Association (Declaration of Helsinki)].

### Apparatus and stimuli

**Figure 1:**
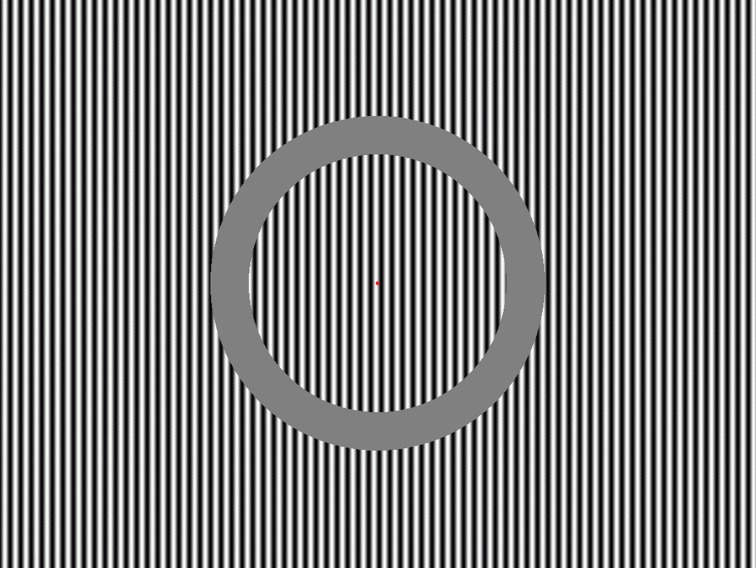
**Schematic view of the stimulus configuration**.

Stimulus presentation programs were created using Matlab (Math Works, Inc.). Stimuli were generated with Cambridge ViSaGe visual stimulus generator (Cambridge Research Systems Ltd., Rochester, UK) and presented on the monitor screen Mitsubishi Diamond Pro 2070SB 22 “(active display area 20,” frame rate 140 Hz) which from the viewing distance of 90 cm subtended 27.6° in width and 20.5° in height. The display elements were target and background vertical sine gratings with following parameters: minimal and maximal luminance 0.13 and 128.2 cd/m^2^, respectively; spatial frequency 0.65 c/°; Michelson contrast 99.8%. Around the central fixation point, a round area was separated by a 1.2° gap, forming a target area, which had a diameter of 8.26°. The whole screen area outside the gap served as a background. (Stimulus configuration is schematically depicted in Figure 1). These specific stimulus parameters showed no background effect on the target motion detection in a previous behavioral study Kuldkepp et al. (2011). Based on that, we expect that when the subject is not paying attention to the background, we can study automatic processing of deviant stimuli there. The background was regularly horizontally moving (200 ms motion, 600 ms pause, velocity 1.6 °/s) and an oddball paradigm (85% standards, 15% deviants) was applied there with horizontal motion direction change as a deviant. In the pilot study for this experiment [unpublished data, result have been reported at 5th Conference on Mismatch Negativity (MMN) and its Clinical and Scientific Applications, 2009, in Budapest, Hungary], we found no exogenous effects of motion direction either on vMMN amplitude or latency and therefore, used rightward motion as a standard and leftward motion as a deviant. At the same time the target area was also horizontally moving: each motion trial had duration of 2225 ms (velocity 0.6°/s, equal left-right probability), random inter-stimulus interval (ISI) was 500, 750, 1000, 1250, or 1500 ms.

### Procedure

The subjects sat 90 cm from the monitor screen in a semi-darkened electrically shielded room and were instructed to keep their eyes on the fixation point. In the “Ignore” condition the subjects had to pay attention only to the target area and to respond as quickly as possible to its motion onset by pressing a corresponding button on the response box (i.e., give a simple reaction). In the “Attend” condition, the instruction was to react to the motion onset of the target area, but depending on whether it is moving in the same or opposite direction with the background, press one of the two corresponding buttons on the response box (i.e., make a choice reaction). One experimental session lasted for about 13 min.

### EEG recording and data analyses

Electroencephalography (EEG) was recorded with BioSemi Active Two system (BioSemi, Amsterdam, Netherlands) using 32 active electrodes (placement based on the international 10/20 system). Reference electrodes were placed on ear lobes. To monitor blinks and eye movements, vertical electrooculogram was recorded with electrodes below and above the right eye and horizontal electrooculogram with electrodes at the right and left outer canthi of the eyes. Online recording was done in DC mode with 1024 Hz sample rate and 0.16–100 Hz band-pass filter. Offline data analyses were done using Brain Vision Analyzer 1.05 (Brain Products GmbH, Munich, Germany). The signals were filtered from 1 to 30 Hz (24 dB/octave). Ocular correction was done using a built-in algorithm (Gratton et al., 1983). Artifact rejection was done with following criteria: maximal allowed voltage step 50 μV; maximal allowed absolute difference of two values in the segment 100 μV; minimal and maximal allowed amplitudes −100 and 100 μV; no more than 100 ms of consecutive low activity (0.5 μV). Nine participants' data were excluded from the final analyses due to technical problems with EEG recording or excessive artifacts. As we were interested in the change detection process in two different attention conditions, EEG data for background events were used for the ERP analyses. We extracted epochs of 700 ms duration (including 100 ms pre-stimulus period) around background motion onset to calculate ERPs to standard and deviant events. Deviants that occurred right after another deviant were excluded from the analyses. As a result, the mean number of deviants per subject was 124. Also, only standards that were preceded by other standards (i.e., repetitive standards) were included (the first standard after a deviant event might be considered to be a deviant itself in an oddball paradigm, since the deviant also forms a trace to be compared with, but due to its rarity the trace is not reinforced; Näätänen and Winkler, 1999). The amount of deviants and standards to be compared in the individual recordings was equalized as much as possible by selecting random segments amongst standard events (the allowed difference criterion between the number of deviants and standards was four segments). For most of the recordings, the percentage of random segments was between 16 and 22. Since we did not allow bad intervals, there were also recordings where the random segments percentage was 24, 26, 32, and 58; for five recordings we had to allow bad intervals to get enough standards for comparison. As a result, the mean number of standard events included in the analyses was 124. The selected responses for deviant and standard events were averaged across each subject. In the resulting waveforms, mean amplitude values were calculated for each 25 ms latency window in the 100–400 ms post-stimulus time range for each subject. Difference waveforms (vMMN) were calculated for both recordings of each subject (“Ignore” and “Attend” condition) individually by subtracting the ERP waveform of a standard event from the ERP waveform of the deviant event. In the resulting vMMN waveforms, mean amplitude values were calculated on the same basis as described above. One-Way and repeated measures analyses of variance (ANOVA), paired *t*-test for dependent samples and *t*-test for single sample was used for statistical analyses, the normality of residuals was tested for each comparison.

**Figure 2:**
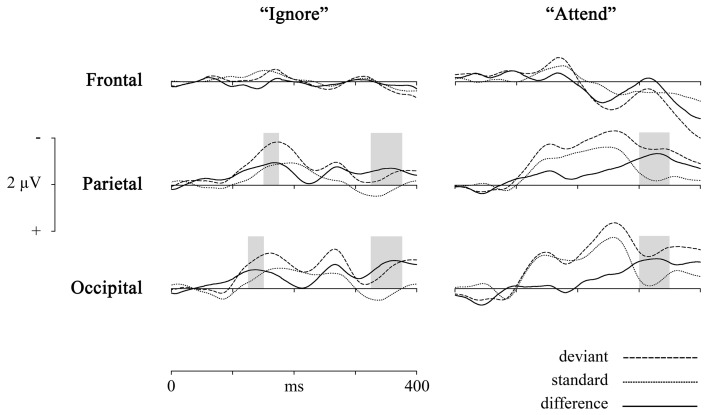
**Group average (*n* = 40) ERPs elicited by deviant (dashed line) and standard (dotted line) events and difference waveforms (deviant—standard, solid line) in 2 conditions (“Ignore,” “Attend”) and 3 scalp locations (comprised of pooled electrodes).** Highest mean amplitudes for difference waveforms are marked with colored bars.

To check if there is no frontal vMMN [as shown for motion stimuli for example by Pazo-Alvarez et al. (2004a)], we pooled together electrodes (AF3, AF4, F3, F4, and Fz) from frontal area [there were no hemispheric differences: in the “Ignore” condition *F*_(22, 934)_ = 0.31, *p* = 0.99; in the “Attend” condition *F*_(22, 934)_ = 1.44, *p* = 0.09] and compared the mean amplitudes of standard and deviant waveforms in all latency windows for “Ignore” and “Attend” conditions. There were no significant differences except for in 3 latency windows in the “Attend” condition [*t*_(39)_ = −2.07, *p* = 0.046 for 225–250 ms; *t*_(39)_ = −2.31, *p* = 0.03 for 350–375 ms; *t*_(39)_ = −3.46, *p* < 0.01 for 375–400 ms latency], the difference wave being positive (as seen in Figure 2) and probably reflecting attention-related P3 component.

To pool the single electrodes together based on their location, we first checked for hemispheric differences in mean vMMN amplitudes for all latency windows in parietal left vs. right regions and found none [in the “Ignore” condition *F*_(22, 934)_ = 0.56, *p* = 0.95, in the “Attend” condition *F*_(22, 934)_ = 1.3, *p* = 0.16], therefore, we pooled all the electrodes in parietal areas together. The electrodes from occipital area of interest were also pooled together. The following two areas were formed for further analyses: Occipital (comprised of O1, O2, and Oz electrodes) and Parietal (comprised of P3, P4, P7, P8, PO3, PO4, and Pz electrodes). Focus on the parietal and occipital scalp areas is supported by previous results (e.g., Pazo-Alvarez et al., 2004a) showing reliable vMMNs for moving stimuli at those locations.

### Behavioral data recording and analyses

For the purposes of within-subjects comparisons we excluded the same nine subjects' data from the analyses that were excluded from the final EEG analyses. The subjects' reactions (the button presses) were online-recorded in ms. For the “Attend” condition, the reactions were also classified to be either correct on incorrect (depending on whether the subject had estimated correctly if target and background area were moving in the same or opposite direction) in the offline analyses. Very fast (<100 ms) and slow (>1000 ms) reactions were excluded from the analyses. To be sure the subjects were participating actively and directing or not directing their attention to the background (depending on the task in hand), we first calculated the hit rates based on target motion trials and subjects' answers. Since the question of interest is how the deviant motion in the background affects reactions to primary task, we included only those trials in the further analyses where both areas (target and background) had been moving together for at least 100 ms and excluded the ones where either or both of the areas were not moving. The differences between RTs were compared by one-way and factorial ANOVA; the normality of residuals was tested for each comparison.

## Results

### Behavioral data

Subjects detected the motion onset of a central target (as indicated by button presses) during 79.6% of all trials in the “Ignore” condition and gave direction estimations on 70.4% of trials in the “Attend” condition. After including only the trials where target and background areas were both moving, mean reaction time (RT) for the “Ignore” condition was 265.2 (*SD* = 116.2) ms. RTs to target motion onset did not differ during standard and deviant background motion: *F*_(1, 1241)_ = 0.78, *p* = 0.38 for 266.5 (*SD* = 115.2) ms and 258.5 (*SD* = 121.5) ms, respectively. In the “Attend” condition, mean RT was 279.2 (*SD* = 131.9) ms, which differed from the mean RT in the “Ignore” condition [*F*_(1, 2928)_ = 8.92, *p* = 0.003]. This is expected since with the number of response alternatives RT increases (Teichner and Krebs, 1974). In the “Attend” condition, there was a significant difference between the RTs in correct vs. incorrect direction estimations [*F*_(1, 1683)_ = 5.54, *p* = 0.02]. Looking into it, we see this difference arises from the trials with deviant motion direction on the background. During standard stimuli, RTs for correct and incorrect answers did not differ: *F*_(1, 1441)_ = 0.46, *p* = 0.50, for 283.6 (*SD* = 136.6) ms and 277.7 (*SD* = 130) ms, respectively. During deviant stimuli, RTs were significantly shorter for incorrect direction estimations [*F*_(1, 242)_ = 5.04, *p* = 0.03), mean RT for the correct answers being 295.8 (*SD* = 139.3) ms and for the incorrect answers 255.3 (*SD* = 125) ms.

### EEG data

Deviant waveforms in Parietal and Occipital areas have a more negative placement compared to standard waveforms in both experimental conditions (Figure 2). Mean amplitudes of standard and deviant waveforms in both areas of interest were compared (repeated measures ANOVA, Benjamini-Hochberg correction applied). The results (Tables 1, 2, Figure 2) show significant differences in early latency windows in both areas for only “Ignore” condition. The highest vMMN mean amplitude emerges in 125–150 ms latency range in Occipital area and in 150–175 ms time window in Parietal area. Significant vMMN amplitudes in later time windows are present in both areas in “Attend” condition starting from around 275 ms and in Occipital area in “Ignore” condition starting from 250 ms. Comparisons (repeated measures ANOVA, Benjamini-Hochberg correction) between “Ignore” and “Attend” conditions in both areas and all time windows separately did not show statistically significant differences, although in the 150–175 ms latency range it was close in both Occipital [*F*_(1,39)_ = 3.2, *p* = 0.09] and Parietal [*F*_(1, 39)_ = 3.03, *p* = 0.09] areas. Analogous tendency was seen in 300–325 latency range in Parietal area [*F*_(1, 39)_ = 3.04, *p* = 0.09].

**Table 1:** **Mean amplitudes of standard, deviant and difference (vMMN) waveforms and repeated measures ANOVA results showing the comparison of standard and deviant mean amplitude for each latency window and condition in Occipital area for 40 subjects**.

**Condition**	**Latency window (ms)**	**Mean amplitudes (μV)**			**ANOVA results**		
		**Standard**	**Deviant**	**vMMN**	***F*-value (39)**	***p*-value**	**η^**2**^ value**
“Ignore”	100–125	−0.03 ± 0.68	−0.31 ± 0.86	−0.28 ± 0.85	4.25	0.046	0.098
	125–150	−0.27 ± 0.89	−0.66 ± 0.95	−0.39 ± 0.85	8.51	0.006^*^	0.179
	150–175	−0.44 ± 0.92	−0.77 ± 0.92	−0.33 ± 0.82	6.48	0.015^*^	0.142
	175–200	−0.45 ± 0.93	−0.61 ± 0.92	−0.16 ± 0.95	1.08	0.3	0.027
	200–225	−0.39 ± 0.79	−0.42 ± 0.99	−0.03 ± 1.02	0.04	0.84	0.001
	225–250	−0.34 ± 0.77	−0.57 ± 1.01	−0.23 ± 1.02	2.11	0.15	0.051
	250–275	−0.36 ± 0.91	−0.87 ± 1.05	−0.52 ± 0.93	12.34	0.001^*^	0.240
	275–300	−0.19 ± 0.85	−0.56 ± 0.97	−0.37 ± 0.86	7.47	0.009^*^	0.161
	300–325	0.11 ± 0.85	−0.19 ± 0.95	−0.29 ± 0.86	4.69	0.04	0.107
	325–350	0.20 ± 0.92	−0.30 ± 0.98	−0.50 ± 0.88	12.84	0.0009^*^	0.248
	350–375	0.07 ± 0.92	−0.53 ± 0.87	−0.60 ± 0.94	16.39	0.0002^*^	0.296
	375–400	−0.06 ± 0.81	−0.62 ± 0.92	−0.56 ± 1.0	12.5	0.001^*^	0.243
“Attend”	100–125	−0.25 ± 0.72	−0.30 ± 0.84	−0.05 ± 0.99	0.12	0.73	0.003
	125–150	−0.67 ± 0.92	−0.72 ± 1.09	−0.05 ± 0.92	0.12	0.73	0.003
	150–175	−0.68 ± 0.93	−0.69 ± 1.02	−0.02 ± 0.85	0.01	0.91	0.001
	175–200	−0.61 ± 0.93	−0.57 ± 0.99	−0.04 ± 0.93	0.09	0.77	0.002
	200–225	−0.68 ± 0.97	−0.83 ± 1.18	−0.15 ± 0.96	0.95	0.34	0.024
	225–250	−0.92 ± 0.98	−1.15 ± 1.3	−0.23 ± 1.06	1.8	0.19	0.044
	250–275	−1.04 ± 0.95	−1.33 ± 1.24	−0.29 ± 1.08	2.89	0.1	0.069
	275–300	−0.57 ± 0.95	−1.02 ± 1.14	−0.45 ± 1.02	7.69	0.008^*^	0.165
	300–325	−0.11 ± 0.87	−0.68 ± 1.14	−0.57 ± 1.02	12.61	0.001^*^	0.244
	325–350	−0.20 ± 0.95	−0.78 ± 1.19	−0.58 ± 1.05	12.02	0.001^*^	0.236
	350–375	−0.36 ± 1.01	−0.86 ± 1.19	−0.51 ± 1.14	7.88	0.008^*^	0.168
	375–400	−0.28 ± 0.97	−0.82 ± 1.12	−0.54 ± 1.07	10.01	0.003^*^	0.204

* Marked probabilities are significant after Benjamini-Hochberg correction allowing for 5% false positives.

**Table 2:** **Mean amplitudes of standard, deviant and difference (vMMN) waveforms and repeated measures ANOVA results showing the comparison of standard and deviant mean amplitude for each latency window and condition in Parietal area for 40 subjects**.

**Condition**	**Latency window (ms)**	**Mean amplitudes (μV)**			**ANOVA results**		
		**Standard**	**Deviant**	**vMMN**	***F*-value (39)**	***p*-value**	**η^**2**^ value**
“Ignore”	100–125	−0.06 ± 0.6	−0.31 ± 0.73	−0.25 ± 0.78	4.2	0.047	0.097
	125–150	−0.24 ± 0.66	−0.63 ± 0.68	−0.39 ± 0.75	10.5	0.002^*^	0.212
	150–175	−0.43 ± 0.67	−0.90 ± 0.66	−0.47 ± 0.82	13.13	0.0008^*^	0.252
	175–200	−0.48 ± 0.72	−0.83 ± 0.81	−0.35 ± 0.99	4.95	0.03	0.113
	200–225	−0.44 ± 0.67	−0.52 ± 0.88	−0.08 ± 1.0	0.27	0.6	0.007
	225–250	−0.26 ± 0.63	−0.34 ± 0.94	−0.08 ± 1.03	0.23	0.63	0.006
	250–275	−0.14 ± 0.72	−0.47 ± 0.98	−0.33 ± 1.02	4.25	0.046	0.098
	275–300	−0.02 ± 0.74	−0.33 ± 0.9	−0.31 ± 0.95	4.32	0.04	0.100
	300–325	0.15 ± 0.72	−0.11 ± 0.84	−0.26 ± 0.85	3.86	0.06	0.090
	325–350	0.18 ± 0.75	−0.16 ± 0.91	−0.34 ± 0.9	5.74	0.02	0.128
	350–375	0.07 ± 0.74	−0.27 ± 0.9	−0.34 ± 1.02	4.36	0.04	0.100
	375–400	−0.06 ± 0.68	−0.27 ± 0.98	−0.21 ± 1.08	1.49	0.23	0.037
“Attend”	100–125	−0.16 ± 0.64	−0.29 ± 0.75	−0.14 ± 0.85	1.03	0.32	0.025
	125–150	−0.51 ± 0.72	−0.68 ± 0.91	−0.18 ± 0.79	1.99	0.17	0.048
	150–175	−0.61 ± 0.71	−0.79 ± 0.93	−0.18 ± 0.8	1.93	0.17	0.047
	175–200	−0.66 ± 0.78	−0.73 ± 0.93	−0.07 ± 0.89	0.22	0.64	0.005
	200–225	−0.74 ± 0.82	−0.88 ± 1.02	−0.14 ± 0.96	0.81	0.37	0.020
	225–250	−0.80 ± 0.84	−1.0 ± 1.14	−0.20 ± 0.99	1.6	0.21	0.039
	250–275	−0.78 ± 0.87	−1.09 ± 1.08	−0.31 ± 0.95	4.37	0.04	0.101
	275–300	−0.49 ± 0.87	−0.95 ± 1.0	−0.46 ± 0.95	9.5	0.004^*^	0.196
	300–325	−0.19 ± 0.79	−0.76 ± 1.04	−0.57 ± 1.01	12.85	0.0009^*^	0.248
	325–350	−0.13 ± 0.81	−0.72 ± 1.12	−0.59 ± 1.03	13.27	0.0008^*^	0.254
	350–375	−0.17 ± 0.85	−0.63 ± 1.12	−0.45 ± 1.08	6.97	0.01^*^	0.152
	375–400	−0.13 ± 0.84	−0.49 ± 1.02	−0.35 ± 1.06	4.48	0.04	0.103

* Marked probabilities are significant after Benjamini-Hochberg correction allowing for 5% false positives.

## Discussion

It is common to stress that our very survival depends critically on being able to perceive the movement of significant objects (e.g., falling tree, running predator etc.) that are approaching us or have otherwise been set in motion by an action or some force. Considering the importance of motion perception, it is not surprising that the visual system is particularly sensitive to it (Palmer, 1999) by developing specialized neurological mechanisms tuned to the fast detection of motion (e.g., Newsome and Paré, 1988). Neurons selective to motion direction that are found in higher levels (layer 4B) of the magnocellular pathway are known for their fast temporal resolution (Livingstone and Hubel, 1988). Also, there is evidence of a pre-attentive, automatic change detection mechanism sensitive to motion direction in the human visual system (e.g., Pazo-Alvarez et al., 2004a). Given that, it is not surprising that there was a stronger deflection in response to an unexpected direction of motion (relative to the regularly directed motion) in unattended than attended situation, the main difference being the emergence of an early vMMN component in the “Ignore” condition that was missing in the “Attend” condition. It is important to note that the difference in standard and deviant stimuli was defined by the direction of motion, not by any other physical attribute of the stimuli. What is surprising is that although deviant and standard stimuli are both quickly detected by our brain, the difference between them is, for some reason, quickly (i.e., during the first couple of hundred ms) processed only during the “Ignore” condition. This is unexpected in the light of previous research (Wei et al., 2002) showing two vMMN components in the attended and an earlier negativity only in unattended condition (but see also Maekawa et al., 2005, who report 2 vMMN components emerging in unattended conditions, although they did not have an attended condition to compare with). It is also well known from studies in auditory modality that MMN should be similarly elicited when subjects direct their attention away or toward the standard and deviant stimuli (for an overview, see Näätänen et al., 2007). Our puzzling result may be caused by an unknown artifact which origin is difficult to trace. However, it is also possible that the results reflect a principal difference between auditory and visual processing. Compared to auditory MMN it took approximately two decades to establish the mere existence of vMMN and one of the probable reasons is a difference between auditory and visual attention. The fact that an early vMMN is not seen in “Attend” condition might reflect the executive attention process in visual modality. Schröger (1997) has suggested that attention affects the encoding of the available sensory information, so it seems possible that when the features of standard and deviant stimuli (i.e., motion direction) are actively processed for conducting a difficult primary task (as was the case in our experiment), the visual top-down attention might suppress the automatic change-detection mechanism responsible for the emergence of vMMN (although there are opposite results, e.g., Kimura et al., 2010, showing vMMN only under attention).

It has been argued (see Czigler et al., 2002; Kimura et al., 2009; Kimura, 2012), that the difference between standard and deviant events near the latency range associated with N1 or the early detection could be mainly due to stimulus-specific refractoriness and not reflect a “genuine” mismatch between stimuli. In other words, because of the different probability of standards and deviants (in our study 85 and 15%, respectively) the level of habituation for afferent neuronal populations responding to differential features of either stimulus (horizontal motion direction in our study) is different and early ERP amplitudes related to deviant stimuli could be larger than for standard stimuli. We can easily eliminate the refractoriness-hypothesis, because exactly the same stimulus configuration and probabilities of stimulus types are used in both attention conditions and there is no significant difference in early processing of standards vs. deviants in the “Attend” condition. Also, Kimura (2012) has suggested that for separating N1 ERP component from the “genuine” vMMN the latter has to be outside the range of a usual N1 peak. The early posterior negativity visible in vMMN waveform in the “Ignore” condition of the current study has the highest mean amplitude between 150–200 ms in Parietal and 125–175 ms in Occipital locations. For motion onset of complex stimulus displays the N1 peak has been found below 150 ms (Kremláček et al., 2004) and Kremláček et al. (2006) report an even larger negative component around 110 ms in a vMMN-eliciting paradigm that is probably N1 (they see differences between standard and deviant stimuli that are interpreted as vMMN starting from 145 ms). Based on these findings we can assume that the early significant difference between standard and deviant responses in the “Ignore” condition (as shown in Figure 2 and Tables 1, 2) is in concordance with the features of vMMN.

In addition, we see a second negative-going difference between standard and deviant events starting from around 250 and 275 ms in both posterior areas in both conditions (although it did not yield statistical significance in Parietal area in “Ignore” condition). This difference waveform has two amplitude peaks in the “Ignore” condition, first one in the N2 time range that has been reported by some researchers to be a “genuine” vMMN (e.g., Czigler et al., 2006; Kimura et al., 2009). In the “Attend” condition, we see a more continuous negative waveform, which would suggest the difference in the N2 time range as well as already in the P3 time range (visible in the deviant and standard waveforms), the latter reflecting task-related activity (Näätänen and Winkler, 1999). We see again that the component associated with automatic deviance detection (here in the N2 latency range) is better separated from latter activity in the “Ignore” condition, which is in concordance with the notion of an attenuated MMN response under focused attention (Näätänen et al., 2007).

When looking at the behavioral results, we see that in the “Ignore” condition there is no difference between participants' reaction times during standard or deviant background motion. We have shown this independence of background motion to target motion onset for the same stimulus configuration in our previous paper Kuldkepp et al. (2011). Interestingly, although the effect of background motion is not visible in behavioral responses, it is evident in the ERP results, meaning that events that do not manifest themselves in our behavior can nevertheless, be noticed and registered by our brain. Hence, we have shown that the discrimination of changes in the unattended visual field is possible for visual complex stimuli.

In the “Attend” condition, we see a somewhat surprising result, namely that in case of incorrect direction estimations RTs are significantly shorter if there is a deviant event on the background. The result that a deviant event facilitates incorrect answers (i.e., subjects make more mistakes) has been shown before (Escera and Corral, 2007). But the result of shorter RTs contradicts many of the previous findings showing prolonged behavioral responses in case of task-irrelevant deviant or novel events (for visual modality see for example Czigler and Sulykos, 2010; for auditory-visual cross-modal paradigm Bendixen et al., 2010; for an overview Escera and Corral, 2007). On the other hand, there are studies showing facilitation effects on performance in case of novel or deviant events on some occasions, for example when the rare events carry ecological importance or some informational content [see Wetzel et al. (2012) and SanMiguel et al. (2010) for auditory-visual paradigms]. One explanation to such results is the enhancement of arousal by stimuli that are motivationally significant, which in turn improves performance or readiness to respond. This notion is also supported by Wetzel et al. (2012) who report the facilitation effect to be larger for (ecologically more significant) novel stimuli than artificial deviants. Chen et al. (2010) have argued that novel or deviant events might draw more attention than frequent standard events, which results in subjects being more confident about their decision and answering more quickly. This explanation is plausible with the decreased RTs, because these results are obtained in the “Attend” condition. The facilitation effect seen in our results can be partly explained by both the arousal component and the attention component of the orienting response. It still remains unclear why the deviant event facilitates only incorrect and not correct answers. For example we can exclude the notion of motion direction being a motivationally significant stimulus (as suggested by studies showing cultural preferences of direction, see for example Spalek and Hammad, 2005) and affecting the performance, because there were no exogenous effects of motion direction (as stated in the Materials and Methods section). The result that deviant events facilitate incorrect direction estimations, needs to be therefore, further explored, because we restricted the analyses of behavioral data to only those trials where there was motion occurring in both central and background area of the display and the number of trials was quite low (although the normality of residuals was controlled).

One might ask if we are sure we have manipulated with subjects' attention effectively enough. We have four arguments to support the positive answer to that question. First, the stimulus configuration was chosen based on previous behavioral results of background and target interaction (Kuldkepp et al., 2011). More specifically, we determined the configuration of central and background visual field partition, where the background motion did not affect the detection of motion onset in the central area. We consider these behavioral results to be a solid ground for designing an experiment with a primary motion detection task in the center to investigate vMMN (elicited by background motion) under ignore conditions. Our current results support this approach since there is a clear difference between “Ignore” and “Attend” conditions for vMMN in early latency windows that is not due to state of refractoriness as explained before. Second, we see a positive amplitude peak in the P3 latency range in Frontal scalp area only in the “Attend” condition, which reflects attention-specific task activity (see Pazo-Alvarez et al., 2003, for an overview of N2b-P3a complex findings in the vMMN research). Third, when we look at the number of target trials and the number of subjects' manual responses, we see a high percentage of answered events in both conditions, which suggests that the subjects were actively participating in the task given to them. For example in the “Attend” condition the task was to estimate if the target and background areas are moving in the same or opposite direction, but due to different time intervals there could have been a situation when the background was stationary during target motion onset. Taking this under consideration the 70.4% answer rate is very high for such a difficult task. Fourth, we see that the mean RT in the “Ignore” condition is in an expected range for a motion onset detection task. For the same stimulus size and velocity the mean RT was 277.9 (*SD* = 74.9) ms in our previous study Kuldkepp et al. (2011). This confirms that the subjects were in fact actively participating in detecting any motion onset and responding to it as quickly as possible.

In the line of research of visual motion perception and psychophysics it is rather common to use experimental paradigms which incorporate the whole visual display area (e.g., Raidvee et al., 2011; Hanada, 2012; for visual evoked potentials see Kremláček et al., 2004). Surprisingly, stimulus configurations extending the entire display are not often reported in vMMN research (except for a stimulus configuration used in several studies by Kremláček and colleagues, see Kremláček et al., 2006; Hosák et al., 2008; Urban et al., 2008), although it would be a reasonable way of eliminating the stimulus location effects caused by discrete stimulus presentations. Importantly, this is the first time to show vMMN to motion direction changes with a display where the sequence of target events is separate from the sequence of standard and deviant events, the latter being continuous. We have therefore, solved two problems that existed in previous vMMN studies using moving stimuli and have been critically raised by Czigler (2007) and Kimura (2012). First, the problem of target events appearing in the same time-sequence with standard and deviant events (e.g., Kremláček et al., 2006), and secondly, the problem of standard and deviant displays being non-continuous [e.g., separated by a blank screen like in Lorenzo-López et al. (2004)].

It has been argued (for an overview, see Kimura, 2012) that in an oddball type of MMN paradigm the more prominent processing of a deviant event could be due to its rareness. New vMMN paradigms with equiprobable stimulus presentation have been shown to be effective for controlling the state of refractoriness (see for example Czigler et al., 2006 and Kimura et al., 2009). Derived from that, future directions with continuous whole-display stimulus configurations should include more equal stimulus proportions. In the line of motion detection research this would also mean including different motion directions instead of only horizontal motion and instead of sine-wave gratings probably a random-dot display [where the orientation of elements in the stimulus display would not play a role, see for example Raidvee et al. (2011)].

In conclusion, we have proposed a stimulus configuration for studying change-detection processes in a typical optic flow pattern and for manipulating with subjects' attention. We obtained two deviant-related negativities that we consider to be vMMN responses in parietal and occipital scalp locations. The first negativity has its peak around 150 ms and is evident only in the “Ignore” condition, and the second emerges in latency windows starting from 225 ms and is more evidently separated from the P3 difference again in the “Ignore” condition in occipital location. We also see that even if the deviant and standard stimulus events do not affect the behavior (as is the case in the “Ignore” condition), our brain is able to process those events automatically.

### Conflict of interest statement

The authors declare that the research was conducted in the absence of any commercial or financial relationships that could be construed as a potential conflict of interest.
